# Fatigue Crack Arrest Induced by Localized Compressive Deformation

**DOI:** 10.3390/ma15134553

**Published:** 2022-06-28

**Authors:** Edú R. Barragán, Ricardo R. Ambriz, José A. Frutos, Christian J. García, César M. Gómora, David Jaramillo

**Affiliations:** Instituto Politécnico Nacional CIITEC-IPN, Cerrada de Cecati S/N Col. Sta. Catarina, Azcapotzalco, Mexico City 02250, Mexico; ebarragang1800@alumno.ipn.mx (E.R.B.); jfrutosmtz@gmail.com (J.A.F.); cjgarcia@ipn.mx (C.J.G.); cmendozago@ipn.mx (C.M.G.); djvigu@gmail.com (D.J.)

**Keywords:** fatigue crack arrest, localized compressive deformation, crack tip, 7075-T651 aluminum alloy

## Abstract

The localized compressive deformation (LCD) effect generated by an indentation process at the crack tip on the fatigue crack growth of the 7075-T651 aluminum alloy is reported. Eccentrically loaded single-edge crack tension specimens (ESE(T)) were pre-cracked at a crack length of about 20 mm by applying a constant amplitude fatigue loading. Subsequently, the LCD process was performed by using a semi-spherical indenter with a radius of 16 mm to compress the crack tip zone at different forces (5.0, 7.0, 12.5, 13.5, 15.5 kN), applied on the opposite surfaces of the specimens. The fatigue cracking process was continued on the compressed samples until an overall crack length of about 30 mm was obtained. The compressive load and the number of delayed cycles is discussed in terms of crack length and crack tip opening displacement (CTOD). A direct relationship between the compressive force induced by the LCD process and the delay of the crack propagation due to the crack arrest was observed. This effect became evident at a compressive force of 5.0 kN, where the crack propagation was arrested for about 9000 cycles in comparison with the non-LCD sample. However, when the force increased, the crack arrest also increased. The crack was considered to be completely arrested at a compressive load of 15.5 kN, since the crack did not grow after the application of more than 3 × 10^6^ cycles.

## 1. Introduction

The 7075-T651 aluminum alloy is widely used for aircraft structural components that require superior strengths. Aluminum alloys used in civil aircraft remain the top engineering material, because the majority of aircraft are made with at least 70% aluminum alloys [1]. Most of these components are subjected to cyclic loading, which can produce the nucleation and propagation of fatigue cracks leading to catastrophic failures. Tajabadi [2] analyzed the failure of a structural component made of 7075-T651 aluminum alloy used to strengthen the center box for the wing connection in an Airbus A-300 aircraft subjected to more than 19,000 flight cycles. He reported several stress concentrator sources for the failure, such as corrosion flaws, scratches produced by careless maintenance and geometry changes, which combined to cause the fatigue cracks’ failure. Several factors influence the fatigue crack growth process, but actual engineering analysis is largely dependent upon a stress intensity factor range. Thus, the level of the cyclic load applied (constant or variable amplitude), as well as the geometry of the component, are the most important. It is worth considering that even if the crack’s propagation stage can be represented by a power law behavior, some characteristics of the materials may retard its growth. For example, Ritchie et al. [3] summarize some mechanisms (crack tip shielding) which can retard or even arrest the propagation of a crack; for example, the beneficial residual stress fields. For instance, work-hardening processes can retard fatigue crack growth depending upon the configuration of the applied loads, and the portion of the material plastically deformed [4,5,6]. The surface displacement field induced by the working process affects fatigue crack growth rate. Zumpac et al. [7] studied the fatigue crack propagation process in a 7075-T651 aluminum alloy after a shot peening process. They found that the plastic deformation at the surface decreases the crack propagation rate in comparison with the non-peened material. Hatamaleh et al. [8] performed two different surface treatments (laser and mechanical peening) to analyze the crack closure, reporting that laser treatment on the surface of the samples reduces the crack propagation rate in comparison with the shot-peened sample. Nevertheless, shot and laser peening are superficial process that affect a thin layer on the surface. Klemenz et al. [9] studied the surface layer characteristics of shot peening. They found that the shot-peening effect is about 170–400 μm from surface.

An additional technique used to arrest or at least retard crack propagation is cold hole expansion. Viveros et al. [10] drilled a hole at the tip of a crack in a CT (compact tension) specimen subjected to cyclic loading. Subsequently, the hole was expanded by a rigid tool to induce beneficial compressive residual stresses and the CT specimen was resubjected to cyclic loading. The cold hole expansion induced a crack closure effect that retarded the fatigue crack propagation. Ambriz et al. [11] reported the influence of variable load spectra for the fatigue crack growth in cold hole expanded specimens, applying periodic overloads and underloads to a baseline cyclic load. They observed a synergic effect between the cold hole expansion and the applied variable cyclic load, which resulted in a reduction of 76% for the fatigue crack growth rate with respect to standard cold-hole-expanded specimens subjected to constant amplitude cyclic loading. Even though methods such as cold hole expansion have been shown to be efficient in arresting crack growth, the change in the component’s geometry from the drill may be a disadvantage in some applications in which the component cannot be drilled.

The aim of this work is to propose new methodologies of work hardening to retard or arrest the fatigue crack growth process with the outmost objective of extending the fatigue life of structural components. A novel procedure is presented to induce a localized compressive deformation (LCD) at the crack tip of fatigue specimens made of 7075-T651 aluminum alloy. The novelty of this method is based on the fact that the geometry of the component is not affected by an expanded hole. This advantage, in addition to the simplicity of the device used for the LCD, means this method in an option when the crack arrest effect needs to be induced. Results are reported in graphs of crack length *a* and crack tip opening displacement (CTOD) versus number of cycles *N*. A beneficial and non-proportional relationship between the crack arrest effect due to the compressive force induced by the LCD process was observed. The fracture surfaces were observed by means of scanning electron microscopy (SEM) to reveal insights of the fracture characteristics associated with the crack arrest.

## 2. Materials and Methods

### 2.1. Microstructural and Mechanical Properties

Commercial 7075-T651 aluminum alloy with dimensions of 2400 × 1200 × 6.35 mm was used. The chemical composition (weight percent) of the alloy is shown in Table 1.

This chemical composition is in concordance with the corresponding ASTM standard B209-14 [12].

The grain structure of the material was observed for the three material directions: longitudinal direction (L), short transverse direction (ST), and long transverse direction (LT). Traditional metallographic preparation was performed by grinding with different sand papers (240, 400, 600 1000 1200 and 2000), and mirror polishing with diamond paste of 3 and 1 μm. The microstructure was revealed by means of an anodizing process. Polished specimens were submerged in a solution of HBF_4_ at 2 % in distillated water. A stainless steel anode was used in conjunction with a voltage of 25 V for 180 s. Microstructural images were taken using an optical microscope with polarized light.

Microhardness, tension and fatigue crack growth tests were performed to determine the mechanical properties of the material. Microhardness tests were performed in specimens with mirror polished surfaces by using a Vickers indenter according to the recommendations of the ASTM standard B578-21 [13]. A load of 1.962 N (0.2 kg) was applied for a dwell time of 10 s. Ten indentations were randomly performed on the three material directions previously mentioned, and the average hardness was reported for each direction.

**Figure 1:**
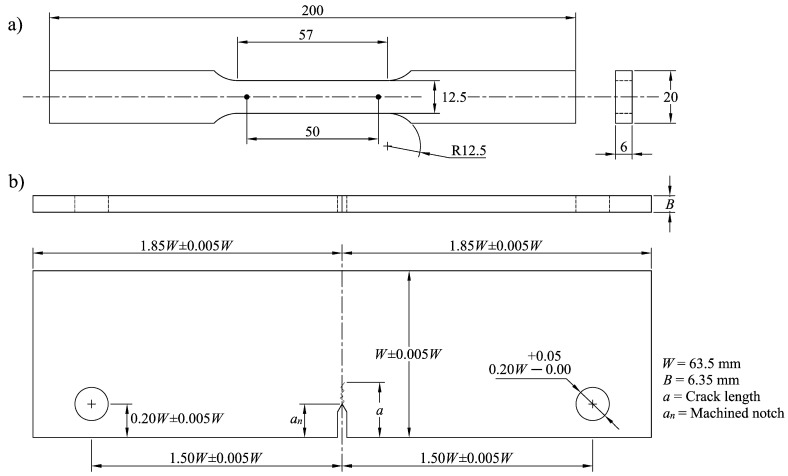
(**a**) Tension test specimen, (**b**) eccentrically loaded single—edge crack tension specimen. Dimensions are in mm.

Tension tests were performed by using specimens oriented along the L and LT directions. The geometry of the standard specimens (Figure 1a) was established according to the ASTM B557M-15 [14]. A servo-hydraulic testing machine equipped with a load cell of 100 kN was used. The tests were performed at a strain rate of 10^−3^ s^−1^. Axial strain was determined from displacement increment measurements by using an extensometer with an initial gage length of 50 mm.

**Figure 2:**
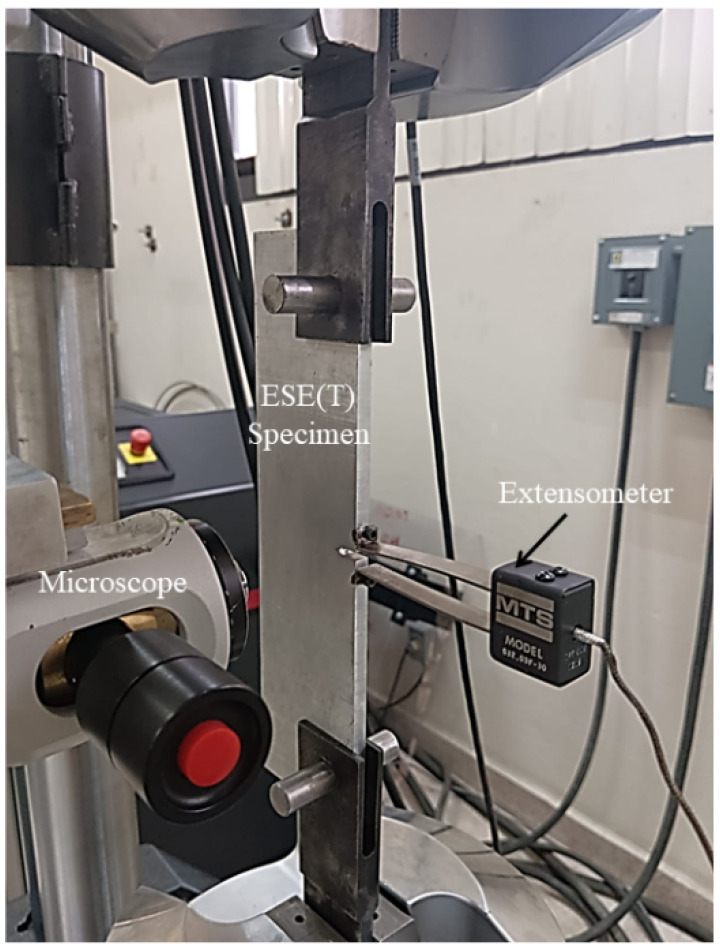
Experimental test setup for the crack propagation.

Fatigue crack growth tests were performed in eccentrically loaded single-edge crack tension (ESE(T)) specimens according to the ASTM standard E647 [15]. The machined notch (12.7 mm) was aligned parallel to the L and LT directions. The tests were performed as shown in Figure 2, by applying a sinusoidal cyclic loading wave (constant amplitude) at a frequency of 30 Hz under ambient temperature.

The load range Δ*P* applied was 5.0 kN with a loading ratio of 0.1. The cyclic load form, load amplitude and load ratio depend on the particular application. In this case, the sinusoidal waveform was chosen since it is one of the most common load forms to represent the constant cyclic amplitude on structural components. The loading ratio of 0.1 was chosen to reproduce a severe amplitude magnitude (tension–tension).

An optical microscope adapted to the fatigue machine with a graduated scale and a resolution of 0.03 mm was used to measure the crack length. The fatigue crack was pre-propagated with the same fatigue parameters mentioned above until it reached a relation of *α* = *a*/*W* = 0.5. Graphs of crack length *a* versus number of cycles *N* were obtained. The fatigue crack growth rate d*a*/d*N* as a function of the stress intensity factor range Δ*K* was plotted according to the experimental data *a*-*N* and Equation (1) [15]:

(1) $$\Delta K=\left[\frac{\Delta P}{B\sqrt{W}}\right]\left\{\alpha^{1/2}\left[1.4+\alpha \right]{\left[1+\alpha \right]}^{-3/2}\right\}F$$

where *B* and *W* are the thickness and width of the specimen, respectively. The shape factor correction *F* is determined by means of Equation (2) [15]:

(2) $$F=3.97-10.88\alpha +26.25\alpha^{2}-38.9\alpha^{3}+30.15\alpha^{4}-9.27\alpha^{5}$$

### 2.2. Localized Compressive Deformation (LCD) in Pre-Cracked Specimens

**Figure 3:**
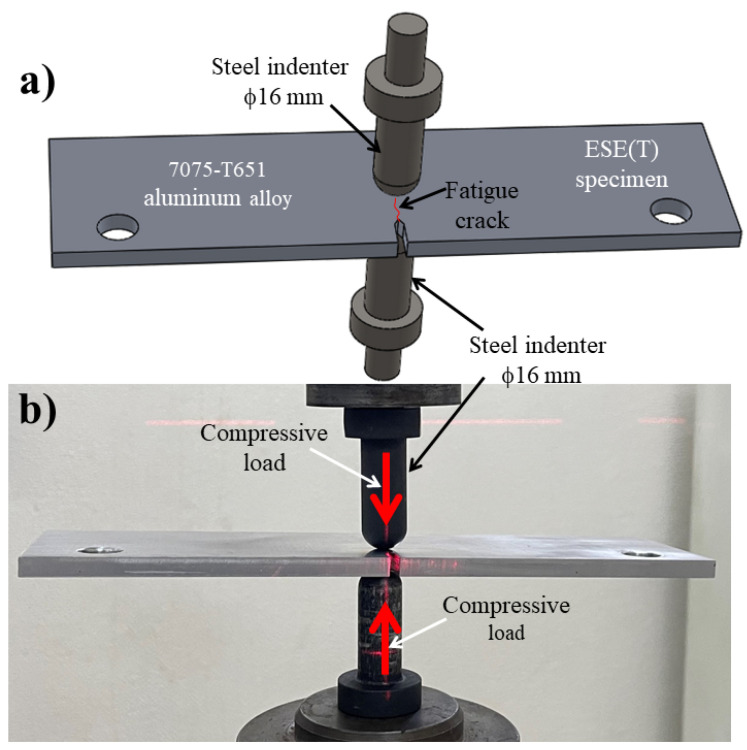
(**a**) Schematic configuration of the LCD process on 7075-T651 aluminum alloy ESE(T) specimen, (**b**) image taken during the LCD process.

A fatigue crack in an ESE(T) specimen of a 7075-T651 aluminum alloy was propagated to reach a ratio of *α* = 0.3 by using the same parameters indicated previously. Subsequently, the LCD process was performed on the surfaces of the specimen at the crack tip (Figure 3). Different values of compressive loads were applied (5.0, 7.0, 12.5, 13.5 and 15.5 kN), by means of semi-spherical opposite indenters attached to the servo-hydraulic machine (Figure 3b). Different compressive loads were considered to determine a relationship between the loading level, the crack arrest effect and the delayed number of cycles.

The fatigue cracking process was continued over the compressed specimens (LCD specimens) by applying the same fatigue crack growth parameters previously mentioned, to reach a relation of α ≅ 0.5. Curves of crack length versus number of cycles are reported.

Crack tip opening displacement (CTOD) measurements were performed to assess the crack arrest effect. These measurements were taken during the fatigue crack propagation, before and after the LCD process. Curves of CTOD versus number of cycles are reported.

### 2.3. Residual Strain Measurements

An approximation of the residual strain condition of the base material and of the specimens with a fatigue crack were determined by means of X-ray diffraction. The following parameters were used: scanning from 30 to 80 degrees with a dwell time of 0.3 s, grid of 1 mm and steps of 0.02. For the fatigue-cracked samples, the analysis was performed focusing the X-ray beam at the crack tip of the specimens. The peak data obtained by the diffractograms were analyzed to quantify the distortion in the diffracted planes (111) and (002). Distortions were measured by considering the full width of the half-maximum (*FWHM*) value of the diffraction peaks. Subsequently, the *FWHM* data were used in conjunction with the Williamson-Hall equation to obtain the average lattice strain *ε* in the diffracted zone [16]:

(3) FWHM·cosθ=4ε·sinθ

### 2.4. Fracture Surfaces

To evidence the effect of the LCD process on the fatigue crack propagation, the fracture surfaces were macro- and microscopically observed. The analyzed samples were subtracted from the specimens with non-LCD and LCD compressed at 12.5 kN. For this compressed load value and above, the crack arrest started to be significant, as will be shown in the measured *a*-*N* and CTOD-*N* curves.

The fracture surfaces of the cracked specimens were microscopically analyzed using a scanning electron microscope. Fracture images were taken from the fracture surface corresponding with the deformed zones. The images were used to observe the fracture surface characteristics and to correlate with the fatigue crack arrest.

## 3. Results and Discussion

### 3.1. Material Characterization

**Figure 4:**
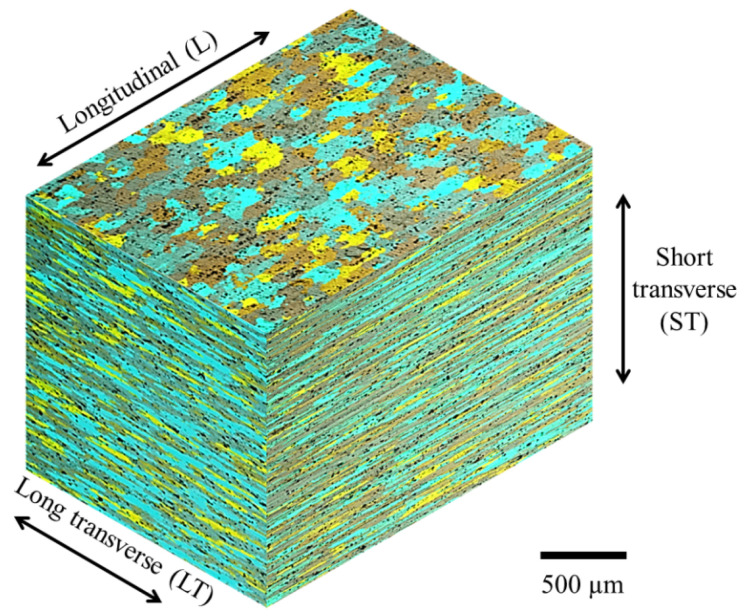
Grain structure of the 7075-T651 aluminum alloy.

The morphology and grain structure of the material is shown in Figure 4. Elongated grains produced by the cold rolling process applied to the material can be observed. Additionally, the presence of intermetallic particles distributed uniformly is observed. In accordance with the alloy system, these particles are aluminum, iron, copper or silicon-rich compounds: Al_12_Fe_3_Si, Al_6_(Cu, Fe) or Al_7_Cu_2_Fe [17,18].

The grain size was estimated with the planimetric method by following the recommendations of the ASTM standard E112 [19]. According to the equation $G=\left(3.322\log N_{A}-2.954\right)$, the average grain size of the 7075-T651 aluminum alloy is about 8.5, 8.9 and 9.6 for the L, LT and ST directions, respectively.

Average results for the microhardness in the material were determined for the three different directions, obtaining 176.6 ± 1.0, 178.7 ± 1.9, and 195 ± 2.5 for the L, LT and ST directions, respectively. Maximum values of hardness were found for the ST direction due to the cold-rolling process effect. However, it is important to note that the principal hardening mechanism in this alloy is the precipitation process produced by the coherent *η*′ phase distributed in the aluminum *α* matrix, according to the following precipitation sequence [20]:

$$SSSS\to GP\;zones\to \eta^{\prime }\to \;\eta$$

where *SSSS* is the supersaturated solid solution, *GP* the Guinier-Preston zones, *η*′ the coherent phase and *η* the equilibrium phase (MgZn_2_).

**Figure 5:**
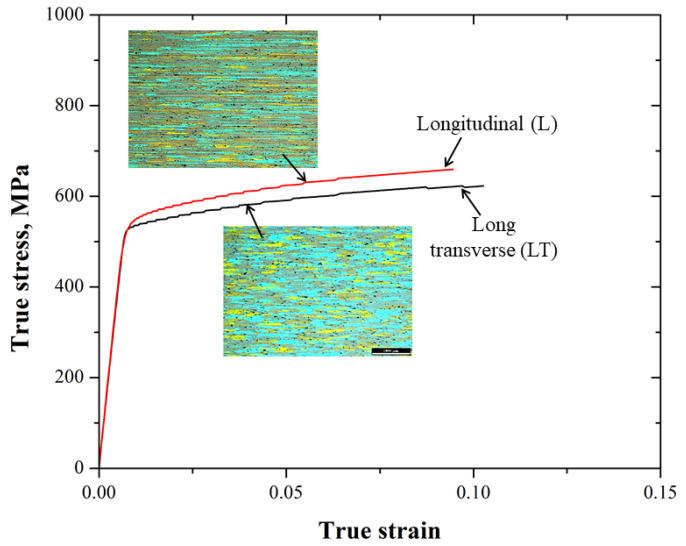
True stress–strain behavior for the 7075-T651 aluminum alloy (longitudinal and long transverse directions).

Figure 5 shows the true stress–strain behavior for the L and LT directions of the 7075-T651 aluminum alloy. A summary of the tensile mechanical properties is shown in Table 2.

As it is possible to observe in Figure 5 and Table 2, the yield and ultimate tensile stress for the L direction tend to be slightly higher than those for the LT direction. This aspect can be attributed to the cold-rolling process and the differences in the microstructure in both directions. Thus, the constitutive equations that represent the true stress–strain behavior are comparable, i.e., they have similar values for the strain hardening exponent *n*.

**Figure 6:**
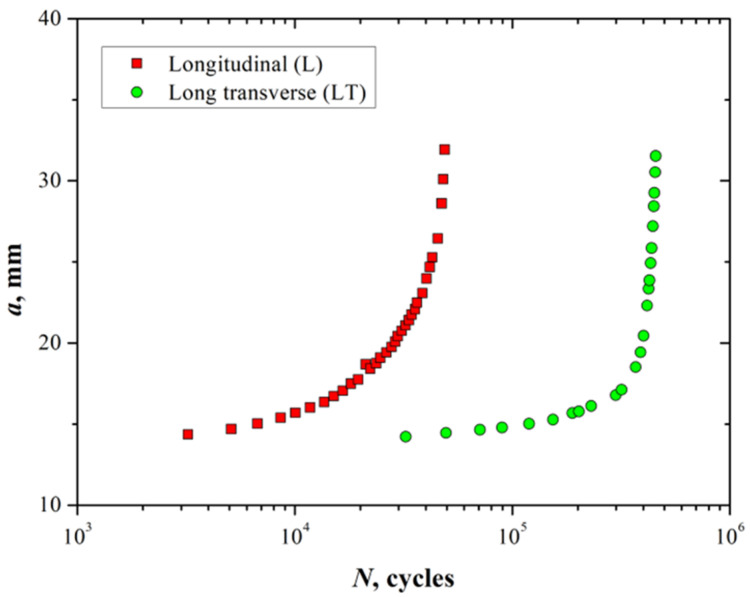
Crack length versus number of cycles for the 7075-T651 aluminum alloy in longitudinal and long transverse directions.

Fatigue crack growth tests were performed to determine the crack growth rate on the L and LT directions of the material. Figure 6 shows the crack length *a* as a function of the number of cycles *N*.

From Figure 6 it is possible to observe that the crack length tends to increase with a lower number of fatigue cycles in the L direction compared with the LT direction. For instance, the number of cycles taken to reach a crack length of 30 mm is about 50,000 cycles for the L direction; however, for the LT direction, the number of cycles applied for the same crack length is roughly ten times more. This aspect can be attributed to the microstructure of the material (Figure 3), i.e., the crack has an advantageous fracture path when the notch is parallel with the L direction of the elongated grains formed by the rolling process. In contrast, when the crack traverses the grains in the LT direction there are more barriers to overcome for the propagation of the crack (grain boundaries), increasing the number of cycles needed to reach a similar crack length compared with the L direction.

**Figure 7:**
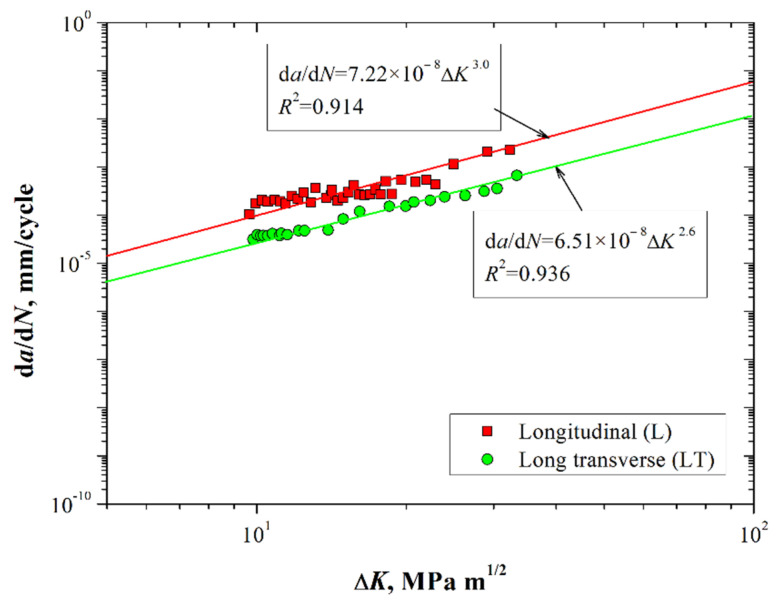
Fatigue crack growth rate as a function of the stress intensity factor range for the 7075-T651 aluminum alloy.

Figure 7 shows the fatigue crack growth rate d*a*/d*N* as a function of the stress intensity factor range *K* for the 7075-T651 material in both directions.

According to Figure 7, it is possible to observe that the slopes from the data fitted to the Paris equation tend to be similar in both directions (*m* values do not have a significant difference). Thus, a similar fatigue crack propagation regimen was presented along the L and LT directions. Fatigue life differences were mostly dependent upon the initial nucleation stage, where microstructural characteristics are relevant. The fatigue tests were performed to determine the crack growth rate conditions in the stable crack propagation region. Thus, considering the *a-N* results (Figure 5) and d*a*/d*N*-*K* (Figure 6), the LCD process at the crack tip was performed in specimens where the notch was aligned along the L direction of the grain structure, i.e., the direction in which the fatigue nucleation stage was smaller.

### 3.2. Localized Compressive Deformation (LCD) in Pre-Cracked Specimens

**Figure 8:**
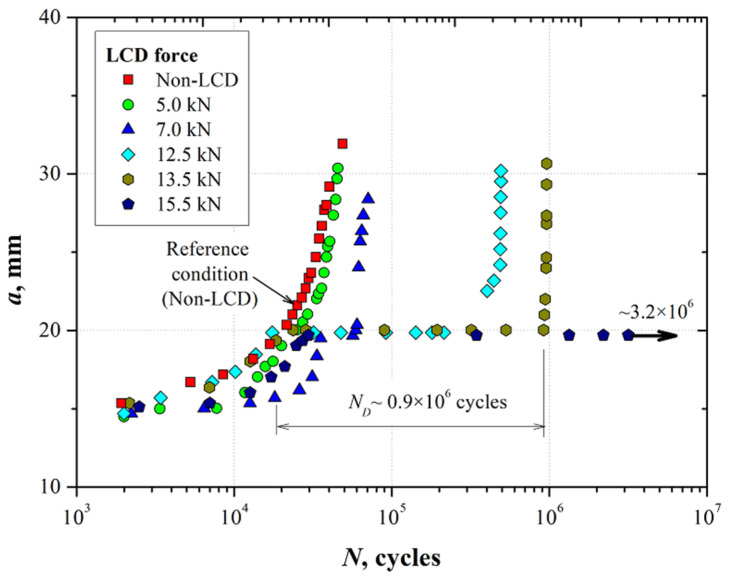
Crack length as a function of number of fatigue cycles for the 7075-T651 aluminum alloy specimens subjected to the localized compressive deformation process at the crack tip.

**Figure 9:**
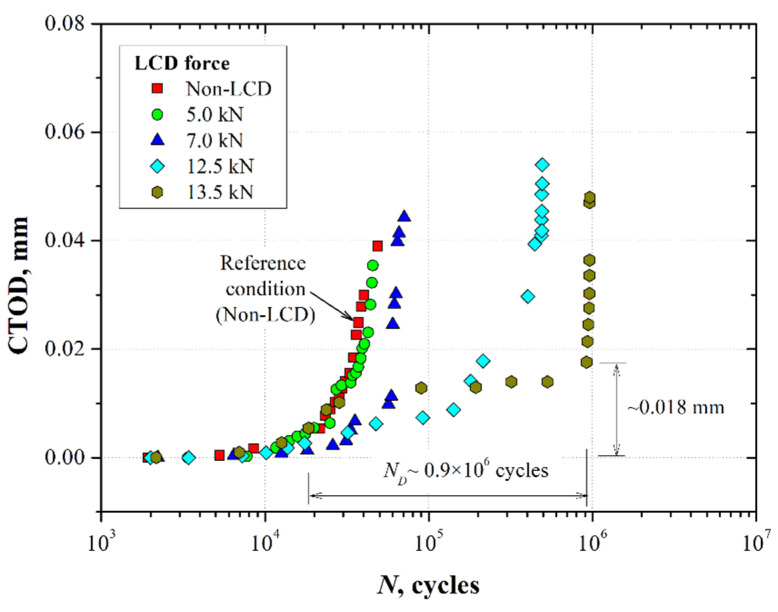
Crack tip opening displacement as a function of the number of fatigue cycles for the 7075-T651 aluminum alloy specimens subjected to the localized compressive deformation process.

Figure 8 and Figure 9 show the results of the crack length and crack tip opening displacement (CTOD) as a function of the number of cycles in specimens pre-cracked by fatigue. These specimens were subjected to the LCD process as indicated in Section 2.2. Subsequently, the fatigue test was continued by applying the cyclic loading conditions specified in Section 2.1. The non-deformed condition (marked as non-LCD) is also included in Figure 7 and Figure 8.

From Figure 8, it is possible to observe a well-defined exponential behavior of the crack length as a function of the number of cycles for the condition with the non-LCD process as well as for the specimen compressed with an indentation force of 5.0 kN, i.e., the LCD process does not produce an evident effect on the fatigue crack growth. In contrast, it has been observed that a fatigue crack arrest effect began to be present when the compressive force at the crack tip was about 7.0 kN. A delay in the number of cycles *N_D_* to reactivate the fatigue crack propagation process was observed. For instance, to grow the crack from 19.5 mm to ~20 mm, *N_D_* was close to 24,000 loading cycles. This effect becomes more marked when the compressive force produced by the LCD process at the crack tip increases. For example, when the compressive force increases to 12.5 kN and 13.5 kN, the number of cycles needed to reactive the fatigue crack propagation process was more than 10 and 45 times higher than the one needed for base material, respectively. Considering the obtained results, the LCD force was increased up to 15.5 kN. For this condition, the crack was arrested at a length of 19.5 mm for more than 3 × 10^6^ cycles.

In general, it has been observed that the LCD process at the crack tip of the pre-cracked specimens in a 7075-T651 aluminum alloy induced a beneficial residual stress field due to the hardening effect, acting as a barrier that delays the crack propagation process. This barrier phenomenon begins to be overcome by the continuous fatigue damage by the cyclic loading, restarting the propagation process and returning to the continuous crack growth behavior of the material.

Since fatigue crack growth in the deformed area generated by the LCD process was not detected, measurements of the CTOD were performed during the fatigue test for the different crack tip LCD forces. The results were plotted as CTOD versus number of cycles (Figure 9).

From Figure 9, it is possible to observe that the LCD effect tended to arrest the crack when the compressive force was higher than 5.0 kN. The fatigue crack propagation decreased when the crack traversed the indented area (Figure 7). In contrast to the results shown in Figure 8 (*a*-*N* graphs), in this case, it was observed that CTOD gradually increased as a function of the number of cycles. This effect was evident when the LCD force increased (12.5 and 13.5 kN), improving the fatigue life of the cracked material. Once the cyclic loading accumulated damage over the residual stress field induced by the indentation area at the crack tip, the CTOD tended to increase suddenly (CTOD~0.018 mm for indentation forces of 12.5 and 13.5 kN). Throughout this process, the increment of the CTOD during the crack propagation through the indented area was promoted by a tunneling effect through the thickness of the sample [21]. This effect could be observed on the fracture surfaces of the samples (Figure 11).

An additional analysis of the *Number of delayed cycles* as a function of the *Compressive load applied* was performed calculating the number of delayed cycles as a function of the LCD force (Equation (4)).

(4) $${\left(Crack\;arrest\;relationship\right)}_{a=20\;\mathrm{mm}}=\frac{Number\;of\;delayed\;cycles}{Compressive\;load\;applied}$$

Since the LCD was performed for *α* = 0.3 (*a* = 20 mm), the crack arrest relationship was calculated only for this value (Table 3). This table shows the number of delayed cycles as a function of the load applied (kN); the calculation was performed for samples where the crack arrest was more evident (7.0, 12.5 and 13.5 kN).

### 3.3. Residual Strain Measurements

**Figure 10:**
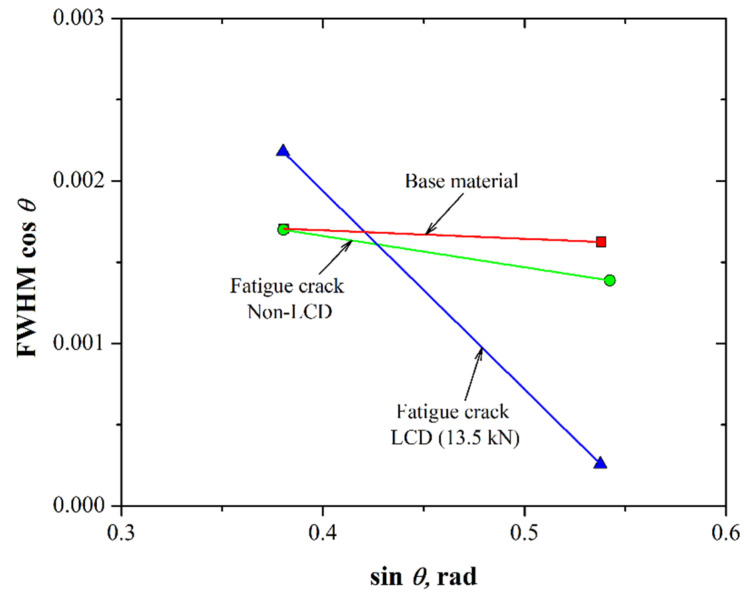
Residual strain approximation obtained from the fitting to the Williamson–Hall equation (Equation (3)) for the 7075-T651 aluminum alloy.

Figure 10 shows the results of the FWHM cos *θ* as a function of the sin *θ* of the diffracted planes (111) and (002) for the base material (without fatigue crack), as well as for an induced fatigue crack and after the LCD process was performed at 13.5 kN.

The fitting of the experimental data to the Williamson–Hall equation allows us to obtain the constitutive equations shown in Table 4 for the 7075-T651 alloy.

According to the constitutive equations, the slope of the diffracted peaks obtained can be used to determine an approximation of the residual strain (Equation (3)). Additionally, if we consider the elastic modulus of the material (~72 GPa), a rough calculation of the residual stress can be assessed. For the base material, the residual strain was about 113 με (~−8.1 MPa); this means that the 7075-T651 aluminum alloy had very low residual stresses due to the stress relief heat treatment given to the material (-T651 condition). The slope from the fitting of the cracked specimen tended to be more negative −488 με (~−35.1 MPa) due to the plasticity-induced closure [22]. On the other hand, it is clear that compressive force performed at the crack tip by the LCD increases the residual strain by approximately 68% with respect to the material without LCD, i.e., the residual strain is about −800 με, which allows us to obtain an approximation of the residual stress of roughly −57.6 MPa. Thus, during the cyclic loading the effective stress applied at the crack tip decreases due to the compressive residual stress, increasing the number of cycles required to reactivate the crack propagation process (Figure 8 and Figure 9).

### 3.4. Fracture Surfaces

**Figure 11:**
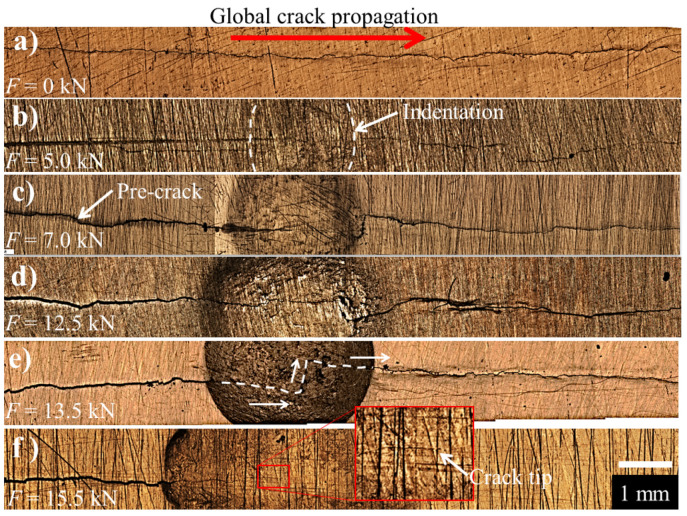
General view of the fatigue crack for the 7075-T651 aluminum alloy, (**a**) base material (non-LCD), (**b**–**f**) different LCD conditions (compressive force from 5.0 to 15.5 kN).

A general view of the crack with non-LCD, as well as for different LCD conditions of the 7075-T651 aluminum alloy, is shown in Figure 11. From Figure 11a, it is possible to observe a regular crack propagation path with a perpendicular orientation with respect to the direction of the applied cyclic loading. In contrast, the semi-spherical indentation produced by the LCD process modifies the global crack propagation (Figure 11b–e), due to the hardening effect produced around the crack tip. This effect induces a localized compressive residual stress field, promoting the crack arrest through the deformed area, which tends to change the cracking load from mode I (opening) to mode III (out-of-plane shear). This phenomenon is clearly evident in Figure 11d,e, i.e., the fatigue crack was arrested for more than 3 × 10^6^ cycles when a compressive force of 15.5 kN was applied during the LCD process (Figure 11f).

**Figure 12:**
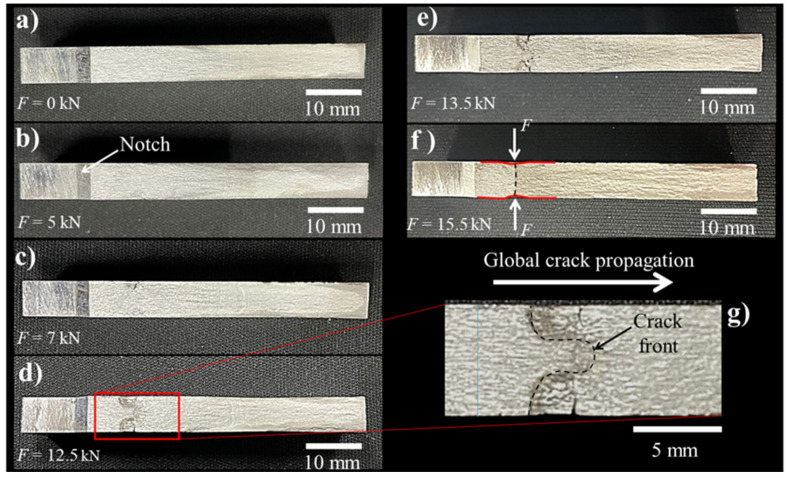
Fracture surfaces of the fatigue crack for the 7075-T651 aluminum alloy, (**a**) base material non-LCD, (**b**–**f**) different LCD conditions (compressive force from 5.0 to 15.5 kN).

Figure 12 shows the fracture surfaces for the different LCD conditions. For the non-LCD (Figure 12a), as well as for the LCD performed at 5.0 kN, a traditional fracture surface produced by the constant amplitude cyclic loading during the stable propagation of the crack is observed, i.e., the crack front tends to be straight. However, when the compressive force used to perform the LCD process increases, the crack tunneling effect becomes evident (Figure 12d,e). To better observe this phenomenon, a section of the fracture surface affected by the LCD process was magnified (Figure 12g). From this figure, it is evident that the crack does not have a straight front. Instead, a semi-elliptical front is favored, promoting tunneling crack growth [21].

**Figure 13:**
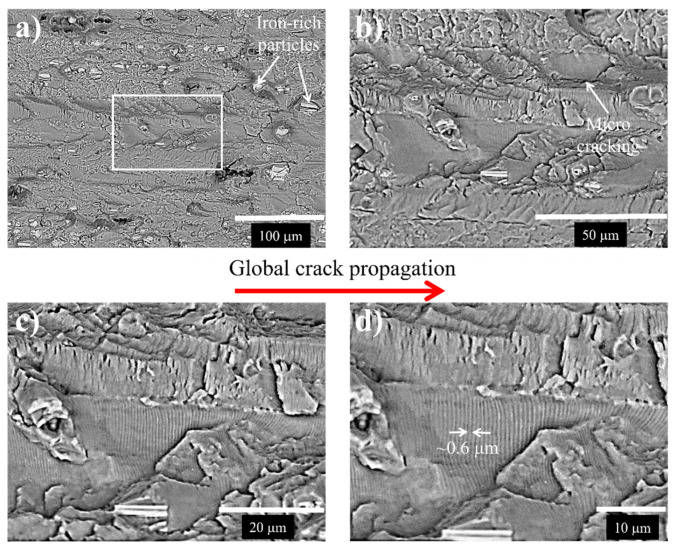
Fracture surface of the 7075-T651 aluminum alloy (base material non-LCD), (**a**) image taken at lower magnification showing Fe-rich particles, (**b**–**d**) details of the rectangle marked in (**a**) at higher magnifications.

**Figure 14:**
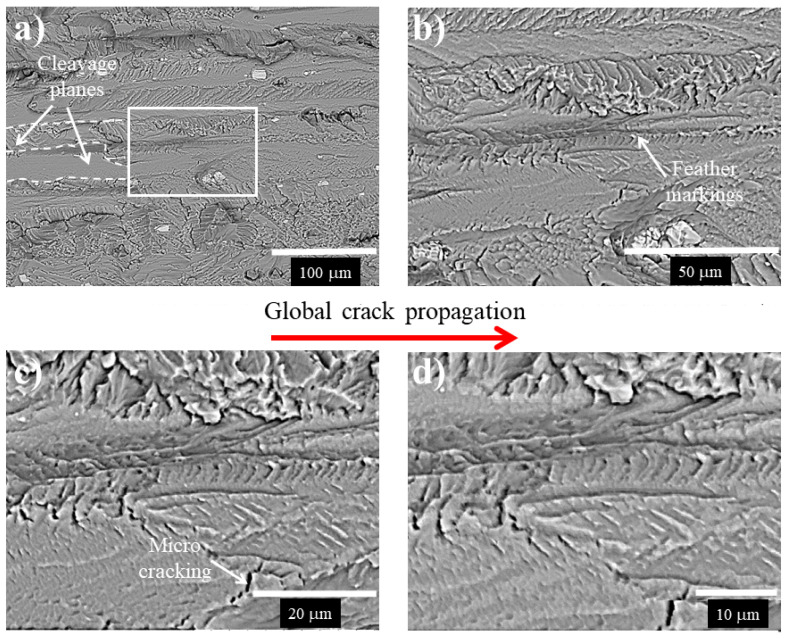
Fracture surface of the 7075-T651 aluminum alloy, after the LCD performed at 12.5 kN, (**a**) image taken at lower magnification showing Fe-rich particles, (**b**–**d**) details of the rectangle marked in (**a**) at higher magnifications.

Details of the fracture surface taken at different magnifications for the 7075-T651 aluminum alloy with non-LCD and LCD performed at 12.5 kN are shown in Figure 13 and Figure 14, respectively.

**Figure 15:**
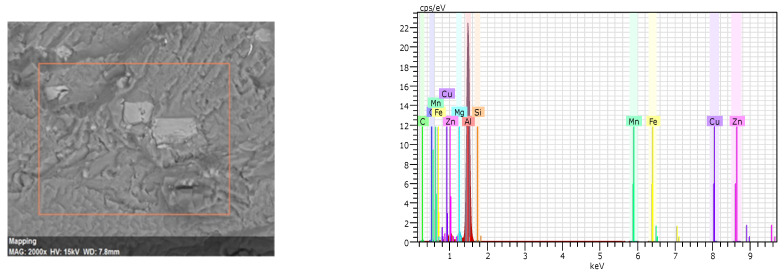
Spectra of the EDS analysis performed on the particles.

The global crack propagation according to perpendicular cyclic loading is indicated (Figure 13 and Figure 14). Brittle particles are observed in Figure 13a and Figure 14a. An EDS punctual analysis revealed that these particles are rich in Fe with some traces of Zn, Si, Cu, Mn and Mg (Figure 15).

According to the EDS analysis, the alloying elements are distributed in the particles as well as the matrix according to the alloy system of the 7075-T651 material.

The observation at a higher magnification of the rectangles marked in Figure 13a and Figure 14a indicates important differences between both fracture surfaces. In the case of the non-LCD (Figure 13a), the characteristic fatigue mechanism has been identified. Striations with a spacing of about 0.6 μm formed during each load cycle were observed (Figure 13b–d). This fracture feature is associated with a continuous crack growth rate during the cyclic loading process.

On the other hand, it is observed that the fatigue fracture surface of the LCD process shows the formation of cleavage planes (Figure 14a), as well as feather markings with no striation formation (Figure 14b–d). These characteristics have been associated with the LCD effect, which produced deformation in the vicinity of the crack tip, changing the local fracture path. According to the literature [22], the cleavage fracture would have perfectly matching faces and be completely flat and featureless. However, in this case, the combination of polycrystalline material with grains randomly oriented (misorientation) with respect to each other, as well as brittle particles and the LCD process, seems to change the mode of cracking load from mode I to mode III. This combination of cracking load modifies the crack front, increasing the energy needed to overcome the out-of-plane shear stress and to re-align the crack front perpendicular to the normal stress. In terms of fatigue cycles, it can take energy of more than one order of magnitude higher, as can be observed in Figure 8 and Figure 9.

## 4. Conclusions

A novel procedure was proposed for promoting the fatigue crack arrest effect in 7075-T651 aluminum alloy ESE(T) specimens. The new process induces a localized compressive deformation (LCD) at the crack tip, which produces favorable residual stress fields due to the hardening effect at the crack tip, increasing the fatigue life of cracked ESE(T) specimens. The crack closure effect is overcome by continuous loading, restarting the fatigue crack propagation process and returning to the continuous crack growth behavior of the material. The LCD procedure with a compressive load of 15.5 kN induced fatigue crack arrest in the ESE(T) specimen, where the fatigue crack did not restart after more than 3 × 10^6^ loading cycles applied. The LCD procedure with compressive loads of 13.5, 12.5 and 7.0 kN also produced the arresting effect, but the fatigue crack restarted after 0.9 × 10^6^, 0.35 × 10^6^ and 0.05 × 10^6^ loading cycles applied. The fatigue crack arrest was not present in ESE(T) specimens subjected to the LCD procedure with a compressive load of 5 kN.

The LCD process does not consider the generation of a hole expansion process at the crack tip of the material to delay the fatigue crack in the material. Instead, a simple indentation in both sides of the fatigue-cracked material at the crack tip is needed. Thus, the LCD process is a suitable option to improve the fatigue life of structural components.

## Figures and Tables

**Table 1:** Chemical composition of the 7075-T651 aluminum alloy (weight percent).

Al	Si	Fe	Cu	Mn	Mg	Cr	Zn	Ti	Others
**88.6**	0.03	0.19	1.7	0.02	2.7	0.18	6.4	0.02	0.16

**Table 2:** Tensile mechanical properties of the 7075-T651 aluminum alloy.

Direction	*E* (GPa)	*σ*_0.2_ (MPa)	*σ_uts_* (MPa)	*H* (MPa)	*n*	Tensile Toughness (MJ m^−3^)
Longitudinal	72	549	600	729	0.07	74.7
Long transverse	72	530	568	794	0.08	64.3

*E* = Elastic modulus, *σ*_0.2_ = yield stress at 0.2% strain, *σ_uts_* = ultimate tensile stress, determined from conventional stress–strain behavior, *H =* strength coefficient, *n* = strain hardening exponent.

**Table 3:** Crack arrest relationship for different LCD forces.

LCD Force (kN)	Crack Arrest Relationship at 20 mm (Cycles/N)
7.0	2.85
12.5	22.4
13.5	66.6

**Table 4:** Constitutive equations obtained from the fitting to the Williamson–Hall equation.

7075-T651 Alloy	Constitutive Equation	
Base material	$FWHM\cos\theta =-1.13\times 10^{-4}\sin\theta +1.70\times 10^{-3}$	(5)
Fatigue crack non-LCD	$FWHM\cos\theta =-4.88\times 10^{-4}\sin\theta +1.70\times 10^{-3}$	(6)
Fatigue crack with LCD (13.5 kN)	$FWHM\cos\theta =-8.00\times 10^{-4}\sin\theta +4.00\times 10^{-3}$	(7)

## Data Availability

The data presented can be available upon request to corresponding author.
